# “I just want them to learn.” The intended role of Teacher’s Book shaped by its writer’s understanding of the local EFL teachers

**DOI:** 10.3389/fpsyg.2022.969403

**Published:** 2022-09-07

**Authors:** Weiying Li, Jianqin Zhang, Zilin Sang

**Affiliations:** ^1^Department of Foreign Languages, Changzhi University, Changzhi, China; ^2^School of Foreign Languages, Shanghai University, Shanghai, China; ^3^School of Foreign Languages, East China Normal University, Shanghai, China

**Keywords:** Teacher’s Book, role of Teacher’s Book, local EFL teachers, ELT materials development, local EFL textbooks

## Abstract

These past few years, programs of local English as a foreign language (EFL) textbook development were launched to adapt to the newly issued English Curriculum Standards in China. They not only develop Student’s Books but also write Teacher’s Books as an integral part of their work. How to write a Teacher’s Book that exactly meets the non-native speaker (NNS) language teachers’ needs was a long-time concern, but few studies have been conducted to address the concern empirically. The present research with a single case design closely examined how a local Teacher’s Book writer’s understanding of the local EFL teachers shaped the role of the Teacher’s Book by looking into the process of an English language teaching (ELT) materials development program in China. It sought to find answers to what the Teacher’s Book writer knew about the local EFL teachers, and how this understanding influenced his conceptualization of Teacher’s Book development. The findings show that the writer’s understanding of local teachers’ conventional teaching practice, and their content knowledge (CK) and pedagogical content knowledge (PCK) play a decisive role in shaping the Teacher’s Book into materials that provide educational affordances to overcome the local EFL teachers’ weaknesses and inject innovation into their conventional practice. These findings have implications for both the international and local ELT materials development programs to compile Teacher’s Book for better local use.

## Introduction

Teacher’s Book (TB thereafter), also referred to as Teacher’s Guide (TG thereafter) (Cox et al., 2019; Tentolouris, 2020), is an important type of curriculum resource in textbook packages. Extensive research has identified the impact of TB on teachers in which it provides curriculum resources and pedagogical support in planning and enacting teaching and scaffolds their reflection (Koljonen et al., 2018; Cox et al., 2019; Sanchez Adorno, 2021). When it comes to innovation in education, TB assumes an even more important role, i.e., providing “educative support” (Jukić Matić and Glasnović Gracin, 2020, p. 2) to inform teachers of the intended curriculum or offer innovative pedagogical support (Lin et al., 2012). In spite of the potential benefits to teachers and teaching; however, it is never a one-size-fits-all solution to teacher needs. In other words, how TB influences teachers to some degree is not only curriculum-specific, but also individually, regionally, and culturally specific (Remillard, 2005). Therefore, it seems sensible and necessary to focus on the process of TB writing within a local ELT materials development program in China.

Developing local ELT materials, especially EFL textbooks, has a long history in China. As early as in the 1920s, EFL textbooks began to be “written, translated, or edited by prominent Chinese authors, educators, translators, or linguists” (Wu, 2015b, p. 31). After the founding of the People’s Republic of China, English syllabi were designed and produced to comply with the country’s political, economic, and educational policies and underwent revisions to cater to newly emerging situations and satisfy the demands in particular times (Adamson, 2004; Liu, 2015b) and the local EFL textbooks kept changing and transferring the intended curriculum. For several decades, textbook development was highly centralized, entrusted to the People’s Education Press (PEP) only, and there was only one set of PEP textbooks based on one syllabus used by middle schools nationwide. In those years, textbooks were the only Student’s Books, and no supplementary materials were developed. In 1988, EFL textbooks began to become diversified with more local-level publishing agencies admitted to join the textbook compiling business (Adamson, 2004) to meet the diverse needs of the growing number of educational situations, “marking the beginning of an era of multiple textbooks based on one syllabus (*yigang duoben*)” (Liu, 2015b, p. 95).

With the development in recent years, well-known university professors have been officially appointed to join the state-approved EFL textbook development programs and take the role of the chief editor responsible for everything in the editorial team. Today, the editorial team not only develop EFL textbooks but also write other supplementary materials, typically TBs, as an integral part of its plan in hopes of making the textbook series competitive among the other state-prescribed EFL textbooks for basic education and more importantly providing support to the local EFL teachers. Writing a TB that provides guidance and support to the local EFL teachers is of great importance in China for two reasons. One is that the TB accompanying a series of EFL textbooks is a key to translating the objectives and the innovative teaching principles, approaches, and methods intended in the syllabi and the new state-approved EFL textbooks. The other is that classroom teachers in basic education cannot choose textbooks freely but “must use whatever is chosen for them by the authorities” (Liu, 2015a, p. 146). The situation means that the TB writer shoulders the responsibility of informing the local EFL teachers as a group of the intended national English curriculum and supporting their practice with TB. However, this is never a simple thing like “I tell you what is there about the curriculum, and you follow it.” Instead, it needs the TB writer to “make difficult assumptions about the existing knowledge of the teachers” (Cunningsworth, 1995, p. 112) so as to ensure that the TBs “meet their users’ needs as fully as possible” (Cunningsworth, 1995, p. 113). In other words, an EFL TB writer must have a sound understanding of the local teachers as non-native English speakers (NNS) to make sound decisions about TB writing. Yet, so far, little has been revealed from empirical studies about how TB writers’ understanding of the local EFL teachers shapes their decision-making about TBs. The study takes a writer-ended perspective to explore the topic by looking into the process of TB writing in an EFL textbook development program in China. The single-case study will theoretically contribute to the existing knowledge about ELT materials development, especially TB writing, by contributing a writer’s perspective. Practically, this study also offers implications for local TB design in other countries and regions.

## Literature review

### Studies of local ELT materials development

English language teaching materials have long been marginalized in the fields of second language acquisition and ESL/EFL language learning and teaching as the research agenda has paid most attention to ESL/EFL teachers and learners (McGrath, 2013). The research on ELT materials development has gained momentum only recently (Abbasian and Hosseinifar, 2015). Yet, most of the literature focuses on exploring systematical principles for ELT materials development (Tomlinson, 2008, 2014; Tomlinson and Masuhara, 2018), evaluation, adaptation, and design (McGrath, 2002) from a theoretical perspective, except for a few empirical investigations (Ulla, 2019; Ulla and Perales, 2021). Among the scant research, attention has been mostly paid to textbooks (Lee, 2015; Syrbe and Rose, 2016; Huang, 2019; Lee and Li, 2019; Rahim and Daghigh, 2019; Atkinson, 2020), but little on the process of writing Teacher’s Book.

An important tendency in the field of ELT materials development is seen in localizing ELT materials (Garton and Graves, 2021b), which involves the adaptation of global ELT textbooks to match the local conditions and context in specific countries and regions (McGrath, 2016). Lopez-Barrios and Debat (2021) classified textbooks into three types: global textbooks (intended for worldwide use by foreign language learners of different ages and language levels), localized textbooks (global textbooks adapted to match a national curriculum and local learners’ needs in a specific country or region), and local textbooks (those specifically designed and produced based on a national curriculum and fit for learners in a specific country or region). A local ELT materials development program develops and produces both localized and local textbooks, either “by ‘experts’ from English-speaking countries, or by local writers, or in collaboration” (Garton and Graves, 2021a, p. 6). The program in the present research produced localized ELT textbooks by local writers but resorted to consultation offered by native speaker experts. The local textbooks are sometimes developed by the local classroom teachers for their own classroom use (Ulla and Perales, 2021), but in the research, they are developed by an officially organized team of local writers for particular EFL classrooms in a region or city.

Localized and local ELT textbooks have been reported to have an advantage over global ones in different ways: complying with the local curriculum (Lopez-Barrios and Debat, 2021), offering learners and teachers familiar content, especially the cultural part (Rahim and Daghigh, 2019; Toledo-Sandoval, 2020), highly related to their real-life world (Garton and Graves, 2021b), more pedagogically adaptive to the local educational environment and practices (Lopez-Barrios and Debat, 2021; Ulla and Perales, 2021), etc. Thanks to the advantages, local textbooks are drawing increasing attention from researchers, but most of their research focuses on content analysis of the product, with little attention spared for the process, and even less for that of TB development.

### Studies of Teacher’s Book

The Teacher’s Book, as an important type of curriculum resource accompanying commercially produced textbooks, plays a role “to assist the teacher to obtain the best possible results from the lessons” (Chitravelu, 1980, as cited in Coleman, 1986, p. 22). It generally assists and supports teachers in the ways of guiding teachers by clearly stating aims and objectives for each lesson, pedagogically scaffolding teachers by providing suggestions on teaching procedures, curriculum resources, and activities pools (Nellutla et al., 2012; Koljonen, 2014; Koljonen et al., 2018) and assuring them by offering answers with further explanations.

Adding to the support observed in the most common ways, TB plays an even more important role as a tool that provides teachers with “educative support” (Jukić Matić and Glasnović Gracin, 2020, p. 2). In this sense, TB becomes a type of teaching material that the TB writers as curriculum developers teach the teachers with and a medium that the TB writer communicates through a voice with teachers (Remillard, 2012). The voice carried by TB just as Lin et al. (2012) noted:

The contents of a guide need to reveal curriculum developers’ concerns, expectations of what teachers are to learn, and attempt to influence teachers’ decision-making…the contents also need to contain several aspects of teacher knowledge, including CK, PCK, and PK. (p. 1003)

That is to say, what TB presents to teachers is not only simple support but also a chance for them to learn and shape their beliefs and knowledge about teaching and learning *via* engaging teachers in practice by following the curriculum developer’s ideas and suggestions. Lin et al. (2012)’s experiment on the effectiveness of the TG to the Nature of Science (NOS) showed that teachers, regardless of NOS-learning experience, were able to improve their students’ understanding of the NOS with the help of TG and were observed to have some change in “their beliefs, knowledge, and intention with regard to integrating NOS into the curriculum” (p. 999). The study indicates that effective TG or its effective use not only provides pedagogical support to teachers but also leads to cognitive improvement in their understanding of content knowledge (CK) and pedagogical content knowledge (PCK). Nellutla et al. (2012) found that the Physical Education Teacher’s Guide in Rwanda introduced not only the knowledge and skills explicitly related to a curriculum or a textbook, but also ergonomics content as an implicit curriculum “with broader aspects of life-skills” (p. 3646). The content was incorporated into classroom activities, potentially raising teachers’ and students’ awareness of the ergonomics of computers and preventing musculoskeletal disorders in children. Jukić Matić and Glasnović Gracin (2020) observed that even though little educative support was prescribed for TG, the educative impact of TG could still emerge *via* teachers’ use of TG in practice.

Another role of TB as a mirror of social discourse was revealed by the text-based analysis research. Critical discourse analysis of the text in TB found that TB was the representation of the discursive discourse in a particular society or culture (Tentolouris, 2020), mirroring the local educators’ beliefs and knowledge about EFL education and practice (Koljonen et al., 2018) and even revealing a conflict between the stated values and goals and implied messages transmitted by its language and structure features (Lechtenberg, 2020). TB’s role as social discourse presenter is indicated more in local curriculum materials.

The limited studies of TB reported above in general disciplines have revealed that TB supports teachers in various ways, and the support becomes even more necessary and important to non-native speakers (NNS) as language teachers in an EFL context due to the fact that “many NNS English teachers feel themselves to be only a hair’s breadth away from where their learners stand, in terms of competence and experience in the target language” (Coleman, 1986, pp. 18–19). However, little attention has been spared to TBs in the field of ELT materials.

In the last century, Coleman (1986) and Cunningsworth (1995) proposed some criteria for TB evaluation based on general discussions. Coleman (1986) stressed the necessity of TB for NNS language teachers when noticing international TBs at that time had failed to guide teachers properly. Based on the recognition, he proposed an instrument with five assumptions for TB evaluation. Coleman suggested that a useful TB should state clearly the basic principles of language and its use, and language teaching and learning; it should give full consideration to the NNS teachers’ ability to explain the target culture, language features, and the ambiguity of content, their ability to understand and carry out the methodology suggested in textbooks, their willingness to deal with open-endedness, etc. Cunningsworth (1995) proposed 22 criteria for TB evaluation based on a discussion of the roles of teachers and TB. The long list of criteria was based on the principle that TB “should meet their users’ needs as fully as possible and should be as flexible as possible” (p. 113). Cunningsworth’s suggestions on the comprehensiveness of TB seemed to be derived from his concern that native-speaker TB writers had to “make difficult assumptions about the existing knowledge of the teachers” (p. 112) around the world.

Kim (2015) closely examined secondary school teachers’ opinions in South Korea and discovered that teachers topped the “comprehensive coverage of information for lesson plans and relevance to the main textbook” (Kim, 2015, p. 6), which included the prediction of learning difficulties and lengthy advice. They showed a moderate preference for the emotion-provoking element, answer keys, explanations of teaching procedures, and language items, but least need for “cultural explanations,” “clear objectives for each lesson,” “guidance on teaching procedures,” “clarity and explicit of approach,” and “information on language items” (Kim, 2015, p. 6). These evaluative opinions indicate the local teachers’ expectations of TB from a user’s perspective.

The literature review demonstrates that studies of TB are limited to a few cases in general disciplines and scant in the field of ELT materials. The existing research has described the roles of TB *via* user-ended investigations or text-based analysis, but no research has been conducted from a TB writer’s perspective yet. The very limited research in the field of ELT materials also puts an emphasis on the consideration of local EFL teachers’ existing knowledge and needs for TB writing. However, no empirical study, yet, explores how a TB writer understands the target EFL teachers’ conditions and needs to make a TB potentially supportive. To bridge the gap, the present research will answer the research questions: What is the TB writer’s understanding of the local EFL teachers? How does the writer’s understanding shape the role(s) intended for the TB?

## Research design

The research adopted a qualitative approach with a single-case study design (Yin, 2014) situated in the most recent local ELT materials development program in China. As Yin (2014) points out, there are five rationales to make the single-case study an appropriate design, i.e., “having a critical, unusual, uncommon, revelatory or longitudinal case” (p.51). Among the five, two rationales of “revelatory” and “longitudinal” formed the conditions for us to make the present research a single-case study design. First, we had “the revelatory case” (Yin, 2014, p. 52). The case in the research was the chief editor of the ELT materials development program in question and we were the key members. As members and researchers, we were deeply involved in the whole process of the local ELT materials development, which rendered us close observers and communicators with the chief editor. The study lasted as long as the program, and in this sense, we had a “longitudinal case” and the time enabled us to collect data of all kinds from different stages of the work.

### The context

#### An overview of English education in China

English language education in China has been undergoing changes, reforms, and innovations and has made great achievements since the implementation of the opening up and reform policy in 1978. During the past 40 years, the English language curriculum went through three general phases of development: it was first brought back on track and modernized from 1978 to the early 1990s when education in China restored order after the turbulence and destruction of the Cultural Revolution, then gradually developed and integrated with the outside world during 1988–2000, and now, it is on the way of deepening reform and innovation since 2001 (Liu, 2015b). Every phase of the English curriculum development was guided by and marked with different syllabi, which are named *English Curriculum Standards* (*ECS* thereafter) today.

The syllabi/*ECS* in China kept introducing and promoting different pedagogies, including grammar-translation method, audiolingual method, structural approach, functional/notional approach, communication language teaching, task-based learning, and eclectic approach through the decades (Adamson, 2004; Wang and Zhang, 2015). As the surrogate motherhood of syllabi, the locally developed EFL textbooks together with TBs always translated the pedagogical approaches either explicitly or implicitly. They were widely used by the EFL teachers in primary and secondary public schools all over the country and intended to guide the teachers to apply and practice the new methods advocated by the syllabi/*ECS* in their classrooms. Consequently, years of English curriculum development have “helped teachers transform their concepts of language teaching, moving from traditional ideas to more modern” (Liu, 2015b, p. 96). Now there are a greater number of teachers with better language proficiency and skills to teach English, and the more effective teaching and learning led to a higher level of English language proficiency in students than ever before.

In spite of the achievements, China’s English education has been facing challenges and one of them is the mismatch between the approaches and methods advocated by the *ECS* and their practices in reality. For example, in the 1970s, communicative language teaching was introduced into English education in China and gained nationwide attention in the 1990s, and then task-based language teaching was encouraged to be used in classrooms since 2000. However, they were found to be ill-adapted to the local context due to factors such as big class sizes, the Chinese examination system, teacher-centered teaching conventions, and a shortage of qualified teachers (Cheng and Xie, 2015; Liu, 2015b). EFL teachers are inclined to traditional teaching in English classrooms adopting elements from the grammar-translation method and audiolingual method. Also, learners are more analytically inclined, preferring to “be told the rules and to learn them by rote” (Liu, 2015b, p. 108).

#### The textbook development program

In December 2014, the Ministry of Education (MOE) launched a new round of high school curriculum reform. The curriculum reform aimed to make a comprehensive revision of *Curriculum Standards for Senior High Schools (Experimental Edition)* implemented in 2004 to integrate the Morality Education put forward on the 18th CPC National Congress into the syllabus. English, as a major course in basic education in China, has been a key area to reform. A revision was arranged, by the MOE, to *English Curriculum Standards for Senior High Schools (Experimental Edition)* issued at the first stage of the deepening reform of the English curriculum in 2003. In 2017, the revision was finished and *English Curriculum Standards for Senior High Schools (2017 Edition)* (*2017 ECS* thereafter) was issued, which was later published in 2018 and slightly revised in 2020.

The *2017 ECS* set up four Core Competencies for English (e.g., language ability, cultural awareness, thinking quality, and learning ability) as new goals for English language teaching and learning in Chinese high schools, updated curriculum content, and formulated standards for academic quality assessment (Ministry of Education, 2017). Accordingly, programs for compiling new EFL textbooks were launched to actualize the spirit in the *2017 ECS*. Since the development of textbooks in China was strictly based on syllabi, and their relationship had changed from one-syllabus-one-textbook to one-syllabus-multiple-textbooks (Liu, 2015a; Wu, 2015a), seven series of EFL textbooks were put on the list for compilation after the *2017 ECS* had been issued to cater to the diversified needs in different parts of China. We took part in one of the textbook compiling programs.

The program was launched in late 2017. It aimed to develop seven volumes of EFL textbooks, each including Student’s Book, Teachers’ Book, and Workbook, for the local senior high schools in a developed region in China. It was, like all the other programs, funded by the local government and organized by the local Educational Bureau. The program required the cooperation of an editorial team with an officially appointed chief editor and a local publisher entrusted by the local Educational Bureau to publish the textbooks. Besides the chief editor, the editorial team was made up of 14 members, including six university English teachers and eight high school English teachers. All the team members have worked as English teachers for 17 to 37 years and most of them have some experience in textbook compiling.

The editorial team was responsible for writing and compiling the textbooks. The first stage of their work was to design and write the Student’s Books. They selected materials, wrote texts, and designed activities for the Student’s Books. The manuscripts of the books underwent four revisions and then were sent to the publishing company for design. Then, they were printed and submitted to the MOE for examination and endorsement. The National Primary and Secondary School Textbook Review Committee (NPSSTRC) under the MOE reviewed the Student’s Books two times in 2019 and 2020. They sent back a report about the merits and demerits of the books every time, and the editorial team revised the textbooks based on the committee’s comments. Finally, they were published for use after the committee’s endorsement. The series of textbooks were listed in the National Textbook Catalog of 2020 as the nationally prescribed EFL textbooks for the local educational administrative departments in a province, region, or city to select textbooks for their high schools.

The second stage of the editorial team’s work continued with the writing of the TB and Workbooks.

#### Process of writing the Teachers’ Book

The process of TB writing involved the action of the initial “designing” of the structure, content framework, and format of the TB, the academically creative “writing” of the texts and activities, etc. (hence, TB design and TB writing are sometimes interchangeably used in the paper). It underwent three phases in general from late 2019 to 2021. In the first phase, the core writers of the Student’s Book wrote the materials for the TB for the respective units. Prior to actual writing, the editorial team held seminars and discussed the structure, principles, and framework of content for the TB, and the chief editor made the decisions. Based on the discussion, a sample unit was written by the chief editor and was shared with all the core writers as a reference. After they submitted the first draft (in Chinese), the chief editor reviewed all their work and made revisions based on their work before submitting them for expert review. In the second phase, after the expert review, the chief editor and two assistant editors began to revise the first draft based on the experts’ revision suggestions. One of the suggestions was to use English instead of Chinese to write the TB. Thus, the second phase of revision involved quite a lot of translation based on the draft, during which the chief editor made further revisions and offered continuous feedback. The third phase started after the second round of expert review in which their suggestions were mainly concerned with the simplification of language and consistency issues.

#### The structure of the Teacher’s Book

The structure of the TB is similar to that of the Student’s Book, encompassing five major sections including Reading and Interaction, Grammar, Listening and Speaking, Writing, and Culture Focus. The TB includes teaching principles, step-by-step teaching suggestions for each task, and the answer keys to the exercises in the Student’s Book. Worksheets for the tasks and the audio and video scripts and keys to exercises in the Workbook are put in the appendix section of the TB. In the two sections with longer reading passages, i.e., the sections of Reading and Interaction and Cultural Focus, additional notes on the author, the passage, and the language are supplied followed by a word study section. In the sections of Reading and Interaction, Listening and Speaking, and Writing, some strategies relevant to reading, listening, and writing were put after teaching suggestions, such as how to take notes in listening, how to write with a topic sentence, and so on. More options of teaching procedures and optional activities were designed for the diverse needs of teachers.

### The case

Chau (pseudonym here) was the officially appointed chief editor taking the leadership for the program with 14 team members and was responsible for every procedure in the textbook design process. He is a well-known university professor with 37 years of teaching experience, and an EFL teacher educator appointed by the local Educational Commission. He enjoys a good academic reputation in the research field of L2 acquisition, EFL teaching, and EFL teacher education. Chau is highly experienced in developing ELT materials and has produced a few sets of ELT textbooks for college students and high school students, which are widely used in China. During the writing process of TB, Chau was responsible for setting up the guidelines, training the team members, who were local English teachers from middle schools and universities, giving comments, making revisions to the team-member-created versions of the TBs, and offering feedback. To a large degree, the work of the TB was based on Chau’s ideas and his understanding of the local EFL teachers.

### Data collection

We collected data throughout the whole process of TB writing from late 2019 to late 2021. We collected them at intervals when it was convenient. We wrote working journals when/after talking with Chau (e.g., working schedule; principles for TB writing; and discussion about how to respond to experts), took notes during the team discussion about the TB design issues (e.g., whether teaching principles should be specified and written for each activity in Student’s Book; what content should be contained in the TB; and which content should be given more attention and which less) or training sessions for team members, and collected different versions of working TBs and other documents such as PowerPoint Slides for teacher training and the *Descriptions of Teacher’s Book Design*. The working TBs were collected as one set of the most revealing data in that there was written feedback in the forms of comments and revisions offered by the chief editor to the team writers and this information informed us of Chau’s beliefs and decisions. During the process of data collection, we made tentative interpretations of the data and generated some initial understandings of Chau’s ideas about TB writing.

To make our initial understanding explicit, we had a 2-h long in-depth interview with Chau at an online meeting due to the COVID-19 pandemic. The in-depth interview was deliberately designed to explore the chief editor’s understanding of TB design, especially his understanding of the local EFL teachers and rationales for designing it. It was developed based on our observations and understanding of his practices during the long-time cooperation. It was a semi-structured interview consisting of two parts, the first including 10 questions about the knowledge and principles of writing TB and the second with 11 episodes in Teacher’s Book 1 and 2 as stimuli to stimulate the chief editor’s ideas and confirm our understanding. The interview was carried out in Chinese and tape-recorded with the chief editor’s consent.

Finally, the data collection formed a final data pool of 15 working journals with about 5,500 words, 3 working versions of the 7 TBs that tracked revisions using Microsoft Word, 2 PPTs, 2 documents, and a 2-h long interview.

### Data analysis

We transcribed the interview, repeatedly read through all the texts collected, listened to the audio, and then conducted a theme analysis of the data. We did initial coding line by line first with the interview transcripts since the intensive and detailed information in it lent itself to “line-by-line coding” (Charmaz, 2014, p.124). Then, we focused on the most frequently occurred codes and used them to “sift, sort, synthesize, and analyze” (Charmaz, 2014, p.138) other datasets, i.e., the working journals, the electronic working TBs, and other materials based on the coding scheme as listed in Table 1. Finally, three themes about Chau’s understanding of the local EFL teachers and one theme about the role he intended for the TB emerged.

**TABLE 1:** Coding scheme for Chau’s understanding of local EFL teachers.

Categories	Subcategories	Codes (samples only)	Sample quotes/Episodes
Understanding of the local EFL teachers’ context	English curriculum	• *English Curriculum Standards for Senior High Schools* • concept of activity-based language learning • key competencies • performance descriptors • viewing as a new skill	*English Curriculum Standards for Senior High Schools* puts forward the concept of English learning activities pointing to the development of subject key competencies, taking activities as the basic organizational form of classroom teaching, and an effective way to cultivate students’ English subject key competencies. (D01Q6)
	Local learning culture	• Learners’ background • enjoying listening to teachers • being shy • different from western learners • not like to express themselves • feel like learning nothing without listening to teachers • practice speaking after class • teacher as a symbol of knowledge	One of the most salient features of Chinese students is that they like listening to teachers. Different from students in western countries, they do not like expressing themselves in class, but prefer to listen to the teacher. (FIL215–217)
	Local teaching tradition	• public schools • unification of teaching • teaching tradition • monolog • enjoying monolog-styled lecturing • script-like lesson plans	EFL teachers in China have an important characteristic: they are accustomed to teacher-centered teaching. They enjoy monolog-styled teaching. (FIL537–539)
Understanding of the local EFL teachers’ CK	Strengths of the local teachers’ CK	• basic communicative ability in English • ability to use the target language • ability to teach in English • having basic English language knowledge • good pronunciation	This is a great progress that has been achieved in English language teaching in the recent 20 years in China. Twenty years ago, many EFL teachers were not able to teach in English. (FIL74–75)
	Weakness of local teachers’ CK	• insufficient literature knowledge • insufficient meta-language knowledge • insufficient linguistic knowledge • insufficient cultural knowledge • insufficient academic knowledge	Language in literature is valuable for EFL learning, and the ability to appreciate and analyze literature are the foundation of literacy…Our teachers do not have enough literature knowledge, nor sufficient literacy in English. (FN201911)
Understanding of the local EFL teachers’ PCK	Strengths of local teachers’ PCK	• having the basic skill to teach English grammar • able to organize activities in the classroom • teacher-centered teaching • monolog • lecture in appropriate opportunities • teaching with preparation skills • teaching with prescribed questions and answers • eliciting prescribed language points	Teacher-centered is not a bad thing. Teachers need to be prepared before class and the language points do need explaining. (FN201902)
	Weakness of local teachers’ PCK	• overdoing teacher-centered teaching • overdoing monolog • lecture • overemphasizing teaching with preparation • teaching with prescribed questions and answers • insufficient open activities • seeing open activities as show • inadequate understanding the pedagogical role of open activities • tending to rush to finish in lecture • cramming in a lot of things • abusing their power in the classroom	They are not good at using open-ended activities in the classroom. They sometimes use open-ended activities in the classroom, but do not know how to answer students’ questions with meta-language. (WJ12P1)

As shown in Table 1, Chau’s understanding of the local EFL teachers included three aspects: their working context, CK, and PCK. The only one theme about the intended role of the TB was “I just want them to learn” and it subsumed three sub-themes as strategies to realize the intended role: “Give them something to lecture about,” “Behaviors first, cognition later,” and “Give them some eye-openers.” We named this group of themes using “the participant’s exact words” (Merriam and Tisdell, 2016, p. 211) as these words exactly reflected Chau’s intentions and behaviors of TB design, and their nature of “emerging” from data. One question about the intended role that concerned us was, “Is one category complete?” We got a positive answer *via* repeated close examination of the data. We found Chau’s intention to make the TB as an educative material was the most salient and significant theme that recurred through the data, and it was completely judged by the criterion that “there should be a minimum of data that you, as the researcher, have determined was illuminating in understanding the phenomenon but are unable to assign to any category or subcategory” (Merriam and Tisdell, 2016, p. 215).

During the analysis, we followed the constant comparative method (Glaser and Strauss, 1967) to compare data with data and the codes within the interview and in other datasets so as to find the similarities and differences that either test or supplement the early identified ideas, which at the same time formed triangulation.

### Credibility and consistency

We applied two strategies to ensure the credibility and consistency of the research. First, we applied the strategy of triangulation in several ways. We collected multiple sources of data and cross-checked them during data analysis (i.e., data triangulation). We also used the strategy of “triangulating analysts” (Merriam and Tisdell, 2016, p. 245) at the stage of data analysis. The authors independently coded the interview transcripts first, then displayed and compared their codes, and resolved the incongruence in their codes *via* negotiation. Based on the agreed codes, the first author continued with the analysis of the rest data. Besides, we used the strategy of “member checks” (Merriam and Tisdell, 2016, p. 246). We sent the interview transcripts and our preliminary analysis back to Chau and asked him whether our interpretation correctly captured his perspectives. This enabled us to get the participant’s confirmation and make some fine-tuning of our analysis according to his suggestions.

## Findings

We identified three themes related to the understanding of the local EFL teachers that affected the TB writer’s decisions to intend the TB as an educative support to the local teachers, and the intention was found to be realized using three strategies. In this section, we first present the three themes of understanding the local EFL teachers and then explicate the strategies the TB writer utilized to shape the role he intended for the TB.

### Chau’s understanding of the local EFL teachers

Chau’s understanding of the local EFL teachers was found in three aspects, including their working context, their content knowledge (CK), and pedagogical content knowledge (PCK) as EFL teachers.

#### Understanding of the local EFL teachers’ context

Chau, who had grown up in the local educational system, was familiar with the learning culture and teaching tradition in classroom practices. As a veteran EFL teacher and teacher educator, he was acquainted with the different rounds of educational reforms in China and teachers’ responses.

Chau had a good knowledge of the development of EFL education in China. He noted the considerable progress of EFL teaching and the overall qualities of its practitioners. He knew clearly about the modes of teaching practices:

.public schools in China value uniformity in practice. Teachers use the same textbooks and Teachers’ Books; they are encouraged to plan lessons together and share the same teaching modes. (FIL65)

He characterized the local teaching practice as “teacher-centered”: teachers dominating the classroom by lecturing through a lesson, and learners sitting and taking notes.

A very important characteristic is that students in China prefer to listen to teachers in classroom. They enjoy listening but not talking too much…Teachers are symbols of knowledge in their eyes… (WJ201901)

Teacher-centered teaching is the most common to see here. The local EFL teachers prefer to lecture through a lesson, cram a lot of things into their plan and rush to finish them in class. (FIL475–477)

As pointed out in the quotes, Chau had a close observation of the local classroom lives and he understood it neutrally as a tradition of teaching and a culture of learning that both the teachers and the learners “prefer” and “enjoy” in a habitually comfortable way. Chau was aware that the teaching context was not easy for the newly articulated conception of “activity-based teaching” by the *2017 ECS* (Ministry of Education, 2017), but he took it as something both to conform to and challenge when considering the design of the TB.

#### Understanding of the local EFL teachers’ content knowledge

EFL teacher’s content knowledge (CK) has much to do with how much and how well they know about the subject of the English language, which encompasses every aspect such as knowledge about linguistics, literature and the target culture, and the skills of using it. According to Chau, the local EFL teachers had inadequate knowledge of the English language despite their satisfactory proficiency.

Most of the local EFL teachers had basic communicative ability in English…they are able to teach English in English now. (FIL72)

Basically, the EFL teachers in China are locals. As local teachers, they lack enough *meta-language* and *knowledge about meta-language*…They don’t have enough literacy because they only received the most basic English education. (FIL111–114)

To teach novel excerpts in Student’s Book well, teachers need *literature knowledge* to make *literary analysis* and appreciate the language use in them. If they don’t have it, then they won’t be able to teach to the point and will bore the students. (FNTT201902)

(Oral feedback about a task of discussing ‘naming’): Teachers talk about only the meaning of a name, but they are not aware of the *historical* and *cultural meaning* behind it. We have the debate of name vs. reality in China, and in Europe, we have Shakespeare’s famous words, but Plato thinks that the nature of human language is naming. Our high school students actually need these profound *philosophical thoughts*, but our teachers are short of it. (WJ201905)

The above quotes about Chau’s comments on the local EFL teacher’s CK reveal his portrait of a qualified language teacher: Being a fluent speaker of the language is a necessary and basic condition for an English teacher, but never a sufficient one. According to Chau, the local EFL teachers had the basic ability to speak and use the language for communication, they could speak English beautifully as far as pronunciation goes and become good models for learners, and they were able to teach the language by modeling reading, instructing strategies, analyzing texts, making basic explanations of grammar rules and words, etc. However, were not enough to make a qualified English teacher, according to Chau. He was concerned that most local EFL teachers lacked “meta-language,” “knowledge about meta-language,” “pragmatic knowledge,” “literature knowledge,” and “historical and cultural knowledge.” This concern was deeply rooted in his belief that language is not merely a set of sounds, words, and grammatical rules, but an entity with social, cultural, and historical properties. Therefore, he maintained that EFL teachers should be equipped with knowledge of this type to better accommodate the high school students’ independent ability to use English, and mature and profound thoughts.

#### Understanding of the local EFL teachers’ pedagogical content knowledge

English as a foreign language teachers’ pedagogical content knowledge (PCK) is the combination of the content of the English language and pedagogy, representing their understanding and principled practice of how to organize, present, and instruct English as a foreign language to learners’ interest.

In terms of PCK, Chau pointed out that the local EFL teachers followed a strong tradition of a teacher-centered method, where they preferred lecturing, but their ability to conduct open-ended activities was quite limited.

The teachers are short of knowledge and skills about open-ended tasks and they have difficulty operating the activities appropriately…They think conducting open-ended activities in classroom is just like “making a show,” only useful for the public open class and the students are not learning but playing. (FIL446–451)

The teachers usually write very detailed lesson plans, so they can lecture through the lesson. But they don’t have enough activities in hand, nor do they know why and how to teach English with open-ended activities. (FNTT201910)

Chau’s comments show that the local teachers’ limited ability to teach English with activities is possibly accountable for several reasons. The first is the teacher’s preference for the traditional teacher-centered practice. The second is their misunderstanding (e.g., “making show” and “playing”) and their ignorance of the rationale, importance, and skills to use activities in the language classroom (e.g., “short of knowledge” and “nor do they know why and how”). The last is their limited repertoire of activities. All of the aspects about the local EFL teachers’ inability to use open-ended activities in language classrooms deeply concerned Chau, because he had a firm belief in the important roles of activities in language learning and teacher qualification.

In summary, Chau held a comprehensive understanding of the local EFL teacher’s contextual factors, strengths, and weaknesses of their CK and PCK, but he focused more on the insufficiency of these aspects and believed that EFL teachers needed support to improve their beliefs and practice about English language teaching. This exerted the most shaping power over his decisions on TB design.

### “I just want them to learn.” – The intended role of Teacher’s Book by Chau

“I just want them to learn.” Chau’s words articulated his intended role of TB as a provider of educative support for the local EFL teachers. Chau stressed the importance of anticipating the local teachers’ needs, which partly lies in his judgment of their knowledge (or lack of it) and partly in their practical conventions (or innovative possibilities). His argument was that every time a teacher uses a new textbook, it is a chance for them to develop their new teaching skills. Therefore, they need to rely on their existing knowledge on the one hand and venture into the unknown territory of practice on the other.

As for how to offer support catering to the needs of the local EFL teachers, we have identified three strategies employed by Chau.

#### Strategy 1: “Give them something to lecture about.”

Chau acknowledged the time-honored tradition of teacher-centered teaching in the form of lecturing in the classroom as something necessary as learners need to be taught about the language in the EFL context, where language input is insufficient for acquisition. He argued that there was no problem with lecturing, but with what they lecture about. In other words, the pervasive teaching practices of translating and modeling reading the text, explaining the meaning of words, or making sample sentences are far from enough to teach a language. Based on the understanding, Chau decided to include the meta-language, literature knowledge, cultural knowledge, and so on into the TB to give the local teachers something to lecture about.

As shown in the quote below, Chau was concerned to incorporate “meta-language” and “knowledge about meta-language” into the TB as he saw linguistic knowledge as an integral part of language teachers’ ability. He believed that the inclusion of the meta-language would empower the local EFL teachers with explaining ability to make the language knowledge and phenomena explicit to learners. Behind his decision to include meta-language was Chau’s belief that the adult EFL learners need explicit learning of English as a foreign language, and his concern that the explicit learning might fail if the local EFL teachers were not equipped with the ability to make explanations about the language features in terms of language phenomena.

As Teacher’s Book designers, we must provide teachers with meta-language and knowledge of meta-language…not only point out what language knowledge it is, but also describe the language phenomena, making clear particular language features of English…If they don’t have the language to describe and explain language phenomena, students would feel confused and make mistakes. (WJ201902a)

Chau also thought it important to include literature knowledge into the TB in addition to the meta-language knowledge because he regarded literature knowledge and linguistic knowledge as the basis of language teachers’ literacy. Literature knowledge, as Chau noted, was indispensable to language teachers. He was concerned that, without sufficient literature knowledge, the EFL language teachers would be unable to make literary analyses of literary works, the language of which was of great value to language learners. Therefore, an amount of literature knowledge was introduced into the sections of TB concerning literary analysis. An example was the analysis of the language features in the excerpt of *The Old Man and the Sea*.

Compelling: …The beginning of the second paragraph of the excerpt is about the appropriate menace of the old man’s “enemy.” The series of sentences continuously use verbs, such as *closed fast, hit the fish, saw his mouth open, drove forward, hear the noise, ripping, threw the fishing spear, crossed with line, and ran straight.* And readers may feel the force of the action when reading this quick-paced description. (TB2, p. 92–93)

The quote extracted from the TB was a demo written to inform the teachers of the language features in the novel and scaffold their teaching. It clarifies the situation (i.e., about the appropriate menace of the old man’s “enemy”), lists language examples, and explains the effect the language might have on readers (i.e., feel the force of actions). The analysis explains how the frequent use of simple language, especially short verbs, creates fast-rhythm motions and tensions and conveys feelings of nervousness to readers. It may function as a demo and a device to support teachers’ learning about literary analysis and give them language to teach in the classroom.

Cultural information was another type of content included in the TB to make the teacher’s lecture overcome the “culture famish” experienced by the high school students, who were already adults with mature and profound thoughts, but most often read simple texts with shallow meaning in the textbooks. Chau insisted that more things behind and beyond the words and the lines should be offered by the teachers to match their students’ complex thoughts. The interpretation of a short quote in a pre-reading activity in the Student’s Book is taken as an example:

As Shakespeare wrote in Romeo and Juliet, “. that which we call a rose/by any other name would smell as sweet.” Like people, places or buildings are also named for many different reasons. In the words of Plato, “For in naming we speak, don’t we? (SB1P36)

Chau stated in the interview that most teachers, to his knowledge, would focus on discussing the meaning of naming in the quote, but likely neglected the profound cultural and linguistic connections in history so as to miss the opportunities to conduct meaningful cultural learning. Considerations like this motivated Chau to write up teaching suggestions like this,

Explain the meanings of the words ‘arbitrary,’ ‘arbitrariness,’ and ‘motivation’ for naming. Naming sometimes is motivated by a good reason, but sometimes is totally arbitrary. (TB1P52)

The extracted suggestions above were followed by specific explanations of “arbitrary” and “arbitrariness” with simplified language. Chau hoped that the suggestions could help teachers understand and explain the quote linguistically so as to add academic, cultural, and linguistic interpretation to their lecture, making language learning more interesting and thought-provoking.

#### Strategy 2: “Behaviors first, cognition later.”

Chau stressed the importance of including detailed suggestions about teaching procedures in the TB to deal with one of his most prioritized concerns: the local EFL teachers’ limited repertoire of open-ended activities and skills to conduct meaning-based language teaching. He highlighted that activities, especially the open-ended ones, play essential roles in providing context for learners to experience and use language meaningfully, which is key to language learning. Hence, he made it important to enable the local teachers to teach by engaging learners in meaning-based teaching activities and understand why and how. He commented that changing their actions should come before their understanding:

Teacher’s Book can not only guide teachers’ cognition about teaching, but also support their behaviors. But I think the latter is more important and should come first… We are not sure that teachers cognitively take in the information we provide as we expect, but they can surely do it right if we offer specific and detailed suggestions to scaffold their teaching behaviors, telling them what to do first, and what next. Then correct understanding will follow successful experiences. (FIL59–63)

Chau deemed the power of TB to shape teachers’ understanding and beliefs in teaching, but he firmly believed it was the first step to forging teachers’ teaching behaviors and making them mediate the progress in teacher cognition.

Based on the logic, detailed, specific teaching procedures for activities, especially for the innovative concepts, were written into the TB as the most important content. The decision was made to transmit the conception of activity-based language teaching advocated by the *2017 ECS* on the one hand, and it was intended to help the local EFL teachers overcome their inadequacy in conducting language teaching *via* open-ended activities on the other. The suggestions on teaching view (i.e., a new language skill prescribed in the *2017 ECS*) were taken as examples.

• Invite Ss to talk about what they think they will see based on the given word list. Refrain from making judgments on Ss’ guesses.
• Play the video with the sound off, and then ask Ss to guess what they have seen and to try to express their ideas in English. Give them language support when Ss have difficulties expressing themselves by writing useful words on the board.
• Play the video with the sound on a few times until Ss have all the answers (TB1, p. 29).

The extracted example is a part of the teaching suggestions given for teaching video-viewing. It typically represents Chau’s intention of teaching the local EFL teachers how to teach the new skill of viewing by guiding them step by step. These steps explicate the exact principle of using video as ELT materials: separating the process of listening and watching, which is totally new to the local EFL teachers. With the suggested teaching procedures and other similar examples in the TB, Chau intended to provide educational affordances to the teachers by scaffolding their practices first and shaping their understanding later. It was his belief that learning teaching procedures were the first step for teacher learning in case they did not fully understand a new conception in a new textbook and syllabus.

#### Strategy 3: “Offer them some eye-openers.”

Diversifying and individualizing the local EFL teacher’s classroom teaching was intended by Chau for the TB in addition to enriching the teacher-centered teaching with content knowledge and scaffolding them with teaching procedures. This strategy was metaphorized by Chau as “offer them some eye-openers”:

We need to offer them some eye-openers. I mean, we can design more options to the teaching suggestions for the typical tasks and write up more optional speaking activities for teacher to choose from, so teachers will realize, “Aha! There are different strategies and methods to teach.” (IT202101)

By offering more options, Chau meant to support teachers with more teaching strategies and activities for them to choose from, experiment with and individualize their teaching, which finally produce an “Aha” effect on the teachers.

**FIGURE 1:**
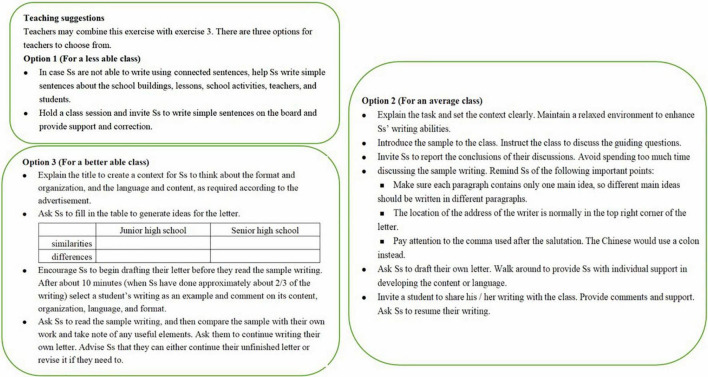
The options for teaching suggestions on a writing section.

These options were designed to help the local EFL teachers to adapt their teaching to their own students’ needs. The teaching suggestions for writing a letter introducing one’s high school life can be taken as an example. The writing task in the Student’s Book was designed to ask students to first analyze a sample letter in terms of its content, language, and editing features and then write a letter. The TB was not designed to follow the order of the tasks in the Student’s Book, but was designed with three different options (see Figure 1).

As demonstrated in Figure 1, the three options differentiate each other in several ways: (1) the students’ language proficiency in Option 1 is the lowest, that in Option 3 is the highest, and that in Option 2 comes between; (2) learners’ independence of operating the writing tasks increases from Option 1 to Option 3; (3) teachers offer most scaffolding in Option 1, but the least in Option 3 and the degree of scaffolding in Option 2 is moderate. All of these features combine into the three optional strategies for the teachers to choose from and adapt to their classrooms, likely making their teaching more individualized and differentiating from others.

In addition to helping individualize the local EFL teachers’ teaching practice, these options are highly informative and educative in several ways: First, implied in the suggested procedures is that writing is an interactive process of meaning-making involving T-S interaction and S-S interaction rather than simply modeling. Besides, it also communicates to the TB users that teaching should always be adaptive to learners’ ability but not just blindly follow the textbooks, and teachers should teach by offering and adjusting scaffolding according to their learners’ performances.

## Discussion

The research with a single case design has explored how a TB writer’s understanding of the local EFL teachers influenced the role he intended for the TB *via* a longitudinal observation of an ELT materials development program in China. The findings show that the TB writer took the TB as a medium providing educative support for teachers (Jukić Matić and Glasnović Gracin, 2020) to overcome the weaknesses in the local EFL teachers’ CK and PCK as he understood and projected innovative elements into their habitual teaching practices. Specifically, the TB writer made the TB an instrument to regulate the local EFL teachers’ cognition and behaviors concerning teaching practice. He incorporated his “concerns, expectations of what teachers are to learn, and attempt to influence teachers’ decision-making” (Lin et al., 2012, p. 1003) into the TB by giving prominence to content selection such as teaching procedures, literature knowledge, meta-knowledge and knowledge about target language culture, etc., which were challenges for the local EFL teachers. The intended role by Chau to the TB is exactly the same as TB was found in other literature as a potential medium to shape teachers’ beliefs and knowledge about teaching and learning (Lin et al., 2012; Nellutla et al., 2012).

The research was significant in that it bridges a gap in the field of TB writing and compiling by contributing a writer’s perspective and looking into the process of ELT materials development in an EFL context. This perspective enables us to understand how a TB writer’s understanding of the local teacher’s knowledge base (either what they have or what they lack) and their conventional teaching behaviors affect his decisions about TB design. It is the first time, to our knowledge, to empirically address Coleman’s (1986) and Cunningsworth’s (1995) concern about writing TB for NNS language teachers.

First, the writer’s knowledge about the local EFL teachers as a shaping tool for TB writing responds to Cunningsworth’s (1995) assertion about the difficulty and necessity to “assume” teachers’ existing knowledge base. While Chau agreed with Cunningsworth (1995) that getting to know the local teachers’ knowledge base is the most challenging part of TB designers’ work, his job showed great significance of it. It was his full understanding of the local teachers’ CK and PCK, to a large degree, that informed him of their merits and demerits, which, in turn, shaped his decisions either to conform to or challenge the local conventional practice. This may assist teachers’ professional development by making a compromise between where they are and where they need to go.

Second, the TB writer’s strategies of designing TB to be educationally supportive address the same issues as Coleman (1986) noted. One of Coleman’s concerns was that the textbook writer’s action of labeling a teaching method (e.g., ‘communicative approach’ or ‘task-based language teaching’) without clarifying exactly how it was implemented was likely to cause confusion and frustration in NNS teachers. To avoid such problems, Chau chose to provide detailed suggestions on teaching procedures, other than make any explanations as Coleman (1986) suggested, for tasks in Student’s Book in hopes of scaffolding the local EFL teachers to conduct task-based language teaching, which has been brought in China’s English education since 2000 but not well practiced in reality. He believed that teachers were likely to change their understanding of a method after successfully experiencing it with the support of procedures. Similarly, the introduction of literature knowledge, meta-language knowledge, and knowledge about the target language culture to the TB are likely to offer great support to the local EFL teachers, who may be unfamiliar with the cultural content and uncertain about particular linguistic content, and lack “the linguistic competence and the self-confidence to elaborate on what the author provides” (Coleman, 1986, p. 27). In addition to introducing the challenging aspects as an educative medium, Chau also highlighted local teachers’ preferences for teaching and learning by providing word lists, adapting content for teachers’ monologs, etc. Designing a TB in such ways was guided by the users-oriented principle, but it was different from Cunningsworth’s (1995) principle by putting the same weight on different aspects in that Chau as a local NNS TB writer knew the local teachers better than the NS TB writers.

It is worth noting, however, that the research suggests that what a TB writer intended may conflict with what TB users expected as revealed by Kim’s study (2015). Specifically, the most prominent content of TB intended by Chau was reported as the least needed by the teachers in Kim’s study. The possible reasons may lie in the different types of support for TB and the roles of teachers. Teachers in Kim’s (2015) study tended to treat TB as supplementary resources for textbooks and classroom lessons and they tended to see themselves as “busy classroom manager” (p. 6), while Chau, as a TB compiler and EFL teacher educator, gave more emphasis on the educative support of TB and expected teachers as learners to internalize the intended new curriculum and improve themselves *via* using the TB. It is like a matter of seeing the same thing from two opposite positions and getting two different pictures. Based on this, it seems understandable that teachers were motivated to use active teaching method *via* the use of less educative TB as observed by Jukić Matić and Glasnović Gracin (2020). Also, the most frequently occurring content in TB may not necessarily reveal the “norms” of teaching practice (Koljonen et al., 2018) in reality but the innovations an educational system is undergoing.

The above discussion further reveals the advantages of the locally produced EFL Teacher’s Book that are similar to those of the localized or local EFL textbooks. Specifically, the locally produced EFL Teacher’s Book by an ELT program in China is closely related to the local teachers’ working life (Garton and Graves, 2021b) due to its writer’s full consideration of the local EFL teachers’ situation. It, therefore, may be congruent with the local EFL teachers’ working context, the tradition and innovation of their teaching practice, the local curriculum, and their leaner’s learning culture and other characteristics (Garton and Graves, 2021b; Lopez-Barrios and Debat, 2021; Ulla and Perales, 2021). It would also be more pedagogically adaptive to the local educational environment and teaching practices (Lopez-Barrios and Debat, 2021) by adding refreshing content to the local EFL teachers’ conventional practice and providing new teaching methods to meet their needs for updating teaching practice and further professional development.

## Conclusion

The research examined how a local TB writer’s understanding of the local EFL teachers shapes the role of TB by looking into an ELT materials development program in China longitudinally. It addressed two questions as to what the TB writer knows about the local EFL teachers, and how his understanding/knowledge influences his decisions. The data analysis reveals that the TB writer had a profound understanding of the local teachers’ teaching context and their strengths and weakness in CK and PCK. The understanding plays a decisive role in shaping the TB writer’s intention and strategies to make the TB as materials that provide educational affordances to overcome their weakness in CK and PCK and inject innovation into their conventional practice.

The findings in the research have several implications. First, the research suggests that NNS TB designers have advantages in their understanding of the local teachers, learners, and culture of learning, but they are challenged by their weaker language ability in some way. Considering this, it is advisable for NS and NNS to cooperate on the compiling work so as to ensure the quality of language and the local adaptability of TB. Also, the employment of local experts in the international ELT materials development program is beneficial to make the materials more locally adaptable and acceptable. Second, the way to determine the role of the TB and the strategies adopted by the TB writer based on his understanding of the local teachers may benefit local TB writing in other regions or countries by scaffolding the writers to reflect before writing: What roles do they want the TB to play in their English curriculum? What are the strengths and weaknesses of the local teachers? What knowledge should be displayed in TB? These questions may help them organize their work more easily. In addition, the conflicts between the intended support and the needs reported by teachers in other literature indicate that teacher training to use a TB is necessary when it is used as a medium to transfer innovative educational conceptions and transform teachers’ behaviors and minds.

Although the research is longitudinal with multiple sources of evidence, it is not without limitations. First, a single case is not enough to make a robust theory, and further research may consider focusing on more participants and programs. Also, it is confined to the single context of China, so the topic deserves further research in different countries and cultures to explore. In addition, since the TB in the research represented the writer’s attempt to support teachers, whether and to what degree it would benefit the teachers need to be further studied, and the match or mismatch between the TB designer’s intentions and its users’ expectations is also a topic to be examined.

## Data availability statement

The original contributions presented in this study are included in the article/supplementary material, further inquiries can be directed to the corresponding author.

## Ethics statement

Ethical review and approval was not required for the study on human participants in accordance with the local legislation and institutional requirements. Written informed consent for participation was not required for this study in accordance with the national legislation and the institutional requirements.

## Author contributions

WL involved in the research design, data collection, data analysis, and writing the first draft of the manuscript. JZ participated in data collection and analysis. ZS involved in the research design, data collection, and data analysis. All authors contributed to manuscript revision, read, and approved the submitted version.
